# Mountain- and brown hare genetic polymorphisms to survey local adaptations and conservation status of the heath hare (*Lepus timidus sylvaticus*, Nilsson 1831)

**DOI:** 10.1038/s41597-022-01794-5

**Published:** 2022-11-03

**Authors:** Craig T. Michell, Jaakko L. O. Pohjoismäki, Göran Spong, Carl-Gustaf Thulin

**Affiliations:** 1 Department of Environmental and Biological Sciences, University of Eastern Finland, P.O. Box 111, FI-80101 Joensuu, Finland; 2 Department of Wildlife, Fish, and Environmental Studies, Molecular Ecology Group, Swedish University of Agricultural Sciences, Skogmarksgränd, 901 83 Umeå, Sweden; 3 Department of Anatomy, Physiology and Biochemistry, Swedish University of Agricultural Sciences, Box 7011, 750 07 Uppsala, Sweden; 4 Present Address: Red Sea Research Center, King Abdullah University of Science and Technology, Box 4700, 23955-6900 Thuwal, Kingdom of Saudi Arabia

**Keywords:** Evolutionary biology, Conservation biology, Genetic variation

## Abstract

We provide the first whole genome sequences from three specimens of the mountain hare subspecies the heath hare (*Lepus timidus sylvaticus*), along with samples from two mountain hares (*Lepus timidus timidus*) and two brown hares (*Lepus europaeus*) from Sweden. The heath hare has a unique grey winter pelage as compared to other mountain hares (white) and brown hares (mostly brown), and face regional extinction, likely due to competitive exclusion from the non-native brown hare. Whole genome resequencing from the seven hare specimens were mapped to the *Lepus timidus* pseudoreference genome and used for detection of 11,363,883 polymorphic nucleotide positions. The data presented here could be useful for addressing local adaptations and conservation status of mountain hares and brown hares in Sweden, including unique subspecies.

## Abstract (summary)

**Table:** 

Measurement(s)	whole genome sequencing
Technology Type(s)	Illumina HiSeq X
Factor Type(s)	Species
Sample Characteristic - Organism	Lepus timidus • Lepus europaeus • Lepus timidus sylvaticus
Sample Characteristic - Location	Sweden

## Background & Summary

Two morphologically distinct mountain hare subspecies are present in Sweden, the nominal subspecies (*Lepus timidus timidus* Linnaeus, 1758), with extensive arctic/subarctic Eurasian distribution, and the endemic heath hare (*Lepus timidus sylvaticus* Nilsson, 1831)^1^, a more temperate adapted subspecies that occur in southern Scandinavia and, presumably, west Estonia^2^. The most conspicuous difference is the colour of their winter pelage, which is white in the nominal mountain hare subspecies and grey in the heath hare.

The geographic distribution of the nominal subspecies has gradually receded northwards in Sweden. One possible reason for this is competition from the introduced brown hare (*Lepus europaeus* Pallas, 1778) in the mid-19^th^ century^3^, but the retreat may also be driven by climate change induced camouflage mismatch or other habitat changes^4–6^. While the heath hare is better camouflaged in areas void of snow, it too might face local extinction due to habitat alteration and competition from brown hare, as indicated from a persistent decline in hunting harvest data^7^.

Earlier research has also shown interspecific gene flow (i.e., introgression) between the two species, *Lepus timidus* and *Lepus europaeus*^8^. Introgression may facilitate adaptation of the non-native brown hare to boreal prerequisites (e.g. seasonal shift to white winter pelage^3,7^, cold climate and/or pathogen resistance^9^). This two-species complex thus offers an excellent model system for exploring how rapid, human-induced ecological change affects species including, their behavioural, demographic and evolutionary interactions over time. In addition to the conservation challenges mentioned, both mountain hares and heath hares are culturally important game species in Sweden and declining populations cause concern.

The genomic data reported here can be used for addressing complex adaptive patterns, assess species interactions and as a resource for developing monitoring tools for threatened populations. They also provide valuable insight into the subspecies differences and evolutionary histories. We have described 11,363,883 polymorphisms in whole genome resequencing data in order to detect differentiating markers and provide an example analysis screening of pigmentation genes that may influence winter pelage colour^10^. In addition to assessment of local adaptations of the two hare species, conservation status of the heath hare and developing management tools, the genomic data reported may also be used for added value to the understanding of the nature of interactions between mountain hares and brown hare in other contacts zones throughout their distribution^3,7,11–13^. In combination with harvest data, pellet inventories and camera trapping, these genetic data with potential additions could be used to develop integrative monitoring tools for mountain hares and brown hares.

## Methods

### Sample collection and DNA extraction

**Fig. 1:**
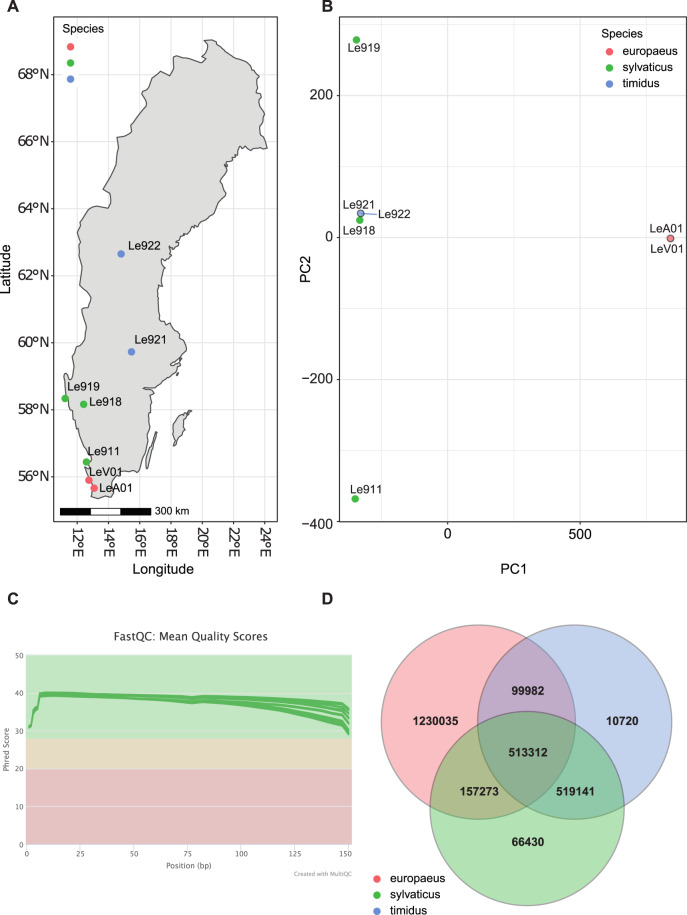
(**A**) Map of sample locations for brown hares (*L. europaeus*; red), heath hares (*L. timidus sylvaticus*; green) and mountain hares (*L. t. timidus*; blue). (**B**) Principal Component Analysis of variants. Overlapping points are outlined in black. (**C**) Phred quality scores across all base for each individual sequencing library. Note, a phred score of Q30 is equivalent to 1 incorrect base in 1000 or 99.9% accuracy in the base call. (**D**) Venn diagram of shared and unique variants by species.

Muscle tissue samples were obtained from seven hares shot from 2005–2017 during the regular hunting season in Sweden. The regular hunting season stretches from September 1 to February 15 for mountain hares in southern Sweden and to the last day of February in northern Sweden. The regular hunting season for brown hares is from September 1 to the last day of February across all of Sweden. The samples are representing two hare species and one hare sub-species (Table 1) in Sweden (Fig. 1A): two individuals of *Lepus europaeus*, two *Lepus timidus timidus* and three of *Lepus timidus sylvaticus*. All three *L. t. sylvaticus* (Le911, Le918 and Le919) samples were collected during January and February, when blue/light grey winter pelage confirm subspecies status (Table 1; Angerbjörn & Flux 1995). In addition, samples from one *L. t. timidus* (Le921) in sympatry with *L. europaeus* was collected in January, when the white winter pelage of this subspecies enable easy species determination. Samples from the final three specimens, one *L. t. timidus* (Le922) and two *L. europaeus* (LeA01 and LeV01), were collected in areas where the respective species occur in allopatry (details available in Table 1). Template DNA was extracted using the QIAsymphony DNA Mini Kit following the manufacturer’s instructions.

**Table 1:** Metadata of the tissue samples (muscle) collected from hares shot during the regular hunting season.

ID	Date collected	Species	Occurrence	Locality	Country	Longitude	Latitude	Sex	SRA accession
Le911	14.1.2017	*Lepus timidus* (*sylvaticus*)	Sympatric	Hallands väderö	Sweden	12.5872	56.443	M	SAMN25691261
Le918	19.1.2017	*Lepus timidus* (*sylvaticus*)	Sympatric	Koberg	Sweden	12.4126	58.1631	n.a.	SAMN25691262
Le919	12.2.2017	*Lepus timidus* (*sylvaticus*)	Sympatric	Hållö	Sweden	11.2276	58.3368	n.a.	SAMN25691263
Le921	10.1.2005	*Lepus timidus* (*timidus*)	Sympatric	Grimsö	Sweden	15.4725	59.7287	F	SAMN25691264
Le922	29.9.2007	*Lepus timidus* (*timidus*)	Allopatric	Svartnäset	Sweden	14.81	62.6502	n.a.	SAMN25691265
LeA01	21.10.2011	*Lepus europaeus*	Allopatric	Alnarp	Sweden	13.0844	55.661	M	SAMN25691266
LeV01	30.10.2011	*Lepus europaeus*	Allopatric	Ven	Sweden	12.7482	55.8995	F	SAMN25691267

Occurrence defines whether the mountain hares and brown hares are sympatric or allopatric in the specific area. Sample Sequence Read Accessions(SRA) are associated with BioProject accession number PRJNA803958.

### Library preparation and sequencing

The seven samples were sequenced over four lanes on a single flowcell on the Illumina HiSeqX. A single Illumina TruSeq PCR free library (Illumina, USA) was prepared for each hare sample at the SciLifeLab in Stockholm, Sweden (National Genomics Infrastructure Stockholm, Sweden). The target insert size of the libraries was 350 bp. The samples were then pooled in equimolar ratios and sequenced over 4 lanes on the Illumina HiSeqX platform using the 2 × 151 Hiseq X SBS chemistry. The Bcl to FastQ conversion was performed using bcl2fastq_v2.19.1.403 of the Illumina software package CASAVA.

### Bioinformatics

Quality of the raw sequencing reads^14^ was assessed using FastQC version 0.11.9^15^ (Fig. 1C). Adapter sequences were then removed from the raw sequencing reads using cutadapt version 2.10^16^ followed by further read quality checks. The trimmed reads were then mapped to the *psedudoreference Lepus timidus* genome^17^ (NCBI accession: GCA_009760805.1) with the Burrows Wheeler Aligner, BWA version 0.7.17^18^ using default the settings for the *bwa mem* algorithm. Each individual was sequenced over different lanes of sequencing, therefore all files for each individual were used as input during the Picard version 2.21.4 markduplicates^19^ step resulting in a single bam file per individual with PCR and optical duplicates removed. Variants were called across the genome in 100,000 bp regions using freebayes version 1.3.1^20^. VCFtools version 0.1.16^21^ was used to apply four rounds of variant filtering to the raw variant file. First, we allowed a maximum missing genotype rate of 50%, a minor allele count 3 and, a minimum variant phred quality score 30. Secondly, we then applied a minor Allele Frequency cut-off of 0.05 and a minimum mean depth of 20. Thirdly, we filtered variants with an allele Balance (AB): AB > 0.25 & AB < 0.75 | AB < 0.01. Finally, a hard filter allowing a maximum mean depth of 30 was applied. This filtering resulted in a variant file with high quality calls. A detailed list of commands and tools can be found in Table 2. Phasing of the genotypes in the final VCF file was achieved using beagle version 4.1^22^.

**Table 2:** Detailed list of the commands used for the dataset generation.

Step	Program	Version	Action	Code example
Preprocessing	Cutadapt^16^	2.10	Remove adapters	for i in $(ls *R1_001.fastq.gz | cut -f 1,2,3,4 -d “_“); do cutadapt -a AGATCGGAAGAGCACACGTCTGAACTCCAGTCA -A AGATCGGAAGAGCGTCGTGTAGGGAAAGAGTGT -o ${i}.P1.fastq.gz -p ${i}.P2.fastq.gz ${i}_R1_001.fastq.gz ${i}_R2_001.fastq.gz; done
Mapping	BWA^18^	0.7.17	Indexing reference	bwa index -r LeTim_1.1_genomic.fna LeTim_1.1_genomic.fna
			Mapping	bwa mem –r LeTim_1.1_genomic.fna P10713_101_S1_L001.P1.fastq.gz P10713_101_S1_L001.P2.fastq.gz
Mark duplicates	Picard^19^	2.21.4	Merge/mark duplicates	picard MarkDuplicates TMP_DIR = /scratch/project_2002674/MICHELLC/FASTQ_RAW/BAMS/tmp I = P10713_101_S1_L001.sortRG.bam I = P10713_101_S1_L002.sortRG.bam I = P10713_101_S9_L007.sortRG.bam I = P10713_101_S9_L008.sortRG.bam O = LE911.sortRGdup.bam M = LE911.marked_dup_metrics.txt
Variant calling	Freebayes^20^	1.3.1	Create regions	fasta_generate_regions.py LeTim_1.1_genomic.fna 100000 > regions.txt
			Call variants	freebayes-puhti -regions regions.txt -f LeTim_1.1_genomic.fna LE911.sortRGdup.bam LE918.sortRGdup.bam LE921.sortRGdup.bam LE922.sortRGdup.bam LE919.sortRGdup.bam LEV01.sortRGdup.bam LEA01.sortRGdup.bam -out hares.vcf
Filtering VCF	VCFtools^21^	0.1.16	Maximum missing genotype 50% -- Minor allele count 3 -- Minimum quality score 30.	vcftools -- vcf hares.vcf -- max-missing 0.5 -- mac 3 -- minQ 30 -- recode -- recode-INFO-all -- out hares.g5mac3
			Minor Allele Frequency 0.05 -- Minimum mean depth of 20	vcftools -- vcf hares.g5mac3.recode.vcf -- min-meanDP 20 -- maf 0.05 -- recode -- recode-INFO-all -- out hares.g5mac3maf05dp20
	vcfutils	0.1.18	Filtering based on Allele Balance (AB): AB > 0.25 & AB < 0.75 | AB < 0.01	vcffilter -s -f “AB > 0.25 & AB < 0.75 | AB < 0.01” hares.g5mac3maf05dp20.recode.vcf > hares.g5mac3maf05dp20AB.recode.vcf
	VCFtools	0.1.16	max mean depth of 30	vcftoools -- vcf hares.g5mac3maf05dp20AB.recode.vcf -- max-meanDP 30 -- recode -- recode-INFO-all -- out hares.g5mac3maf05dp20maxDP30
Phasing SNPs	BEAGLE^22^	5.0	Phase genotypes	java -jar beagle.12Jul19.jar gt = hares.g5mac3maf05dp20maxDP30.recode.vcf.gz out = hares.g5mac3maf05dp20maxDP30.Phased.recode.vcf.gz
Thinning SNPs	VCFtools		Thin SNPs	vcftools -- gzvcf hares.g5mac3maf05dp20maxDP30.Phased.recode.vcf.gz -- thin 500 -- recode--recode-INFO-all -- out hares.g5mac3maf05dp20maxDP30.Phased.thin500.recode.vcf.gz

Details of the final VCF file^23^ were calculated using vt. To calculate the number of shared and unique SNPs, the VCF file was partitioned based on the sample species or subspecies. Then the variable statistics were recalculated using vcffixup version 1.0.0 and filtered to keep only variable sites (AC > 2 and AF < 1)^23^. Finally, the private VCF files were converted to bed files and the intersects of the bed files were calculated using intervene^24^. Population differentiation was estimated in R version 4.0.4 (2021-02-15)^25^ using the *glpca* function contained in the package adegenet version 2.1.5^26,27^.

To generate a mitochondrial phylogenetic tree with other hare species, we assembled the mitochondrial genomes of each individual. The mitochondrial genomes were assembled using NovoPlasty version 4.3^28^. NovoPlasty requires a seed sequence with which to build the assembly from, thus we used the whole mitochondrial genome of *Lepus timidus* (Genbank accession NC024040) as the seed sequence. The assembled mitochondrial genomes were then aligned with those of the *Oryctolagus cuniculus* (accession number AJ001588.1), *L. capensis* (accession number GU937113.1), *L. americanus* (accession numbers NC_024043.1 and KJ397613.1), *L. townsendii* (accession numbers NC_024041.1 and KJ397609.1), *L. coreanus* (accession number KF040450.1), *L. granatensis* (accession number NC_024042.1 and KJ397610.1), *L. tolai* (accession number KM609214.1), *L. yarkandensis* (accession numbers MN450151.1 and MG279351.1), *L. sinensis* (accession number KM362831.1), *L. hainanus* (accession number JQ219662.1), *L. capensis* (accession number GU937113.1), *L. tibetanus* (accession number LC073697.1), *L. europaeus* (accession numbers AJ421471.1 and KY211025.1), and *L. timidus* (accession numbers NC_024040.1, KJ397605.1, KR030072.1, KR030070.1, KR030069.1, KR013248.1 and KR019013.1) using MAFFT v 7.490^29^. The phylogenetic tree was then built using the IQ-tree web service^30^, with the best model determined by ModelFinder^31^ and using 2000 ultrafast bootstrap iterations. A second phylogenetic tree was generated using the genome wide variants identified. This phylogenetic tree was estimated RaXML^32^ version 8.2.12 with a GTR + Γ + ASC model and 200 rapid bootstrapping replicates. The whole genome ML tree was then ultrametricised using the chronos function in Ape version 5.4^33^ to the estimated divergence time of *Lepus europaeus* and *Lepus timidus*.

### Validation

Technical validation of the sequencing quality was performed using FastQC, followed by compilation of the data with MultiQC^34^. To validate the origin of the datasets we used two methods, firstly the confirmation of the winter phenotype and second confirmation of the phylogenetic relationship and their ancestry coefficients. The phylogenetic relationship was assessed based on the mitochondrial genome as described above including other hare species and then using the genome wide variants from the closed analysis. The ancestry coefficients were determined using Poppr version 2.9.3^35^ based on the DAPC method.

### Usage example

To further illustrate a potential use of the dataset, we performed an example analysis of SNPs in 59 known pigmentation genes^10^, which could be used to understand the genetics of pelage color (Fig. 3). First, rabbit or mouse reference sequences were downloaded for each gene contained in Table 2 of Hoekstra, (2006) from the NCBI database. These genes were then mapped onto the *psedudoreference Lepus timidus* genome using the minimap2^36^ splice aware transcript algorithm. We then counted the number of SNPs overlapping the pelage color gene regions identified based on the mapping with BEDtools^37^ and present this data(Fig. 3).

## Data Records

### Sequencing data

The sequencing was successful and resulted in a total of 3.24 billion reads. The sequencing read quality was high with an average phred value greater than Q30 (Fig. 1C). The average mapping rate of the reads to the *pseudoreference Lepus timidus* genome is 93.59% with an average of 79.9% of reads mapping as proper pairs (Table 3). The raw sequencing data^14^ can be accessed from the NCBI short read archive under project accession and individual accession numbers can be found in Table 1.

**Table 3:** Sequencing and mapping statistics.

ID	Read length and type	% Duplicate reads	Error rate %	Reads Mapped (Million)	X Coverage of genome*	% Reads mapped	% Mapped and proper pairs	% MapQ 0	Total sequences (Million)
LE911	150 bp Paired	13.08%	1.70%	463	24	93.40%	79.80%	9.00%	495.7
LE918	150 bp Paired	13.68%	1.68%	441.1	23	93.60%	80.30%	8.90%	471.3
LE919	150 bp Paired	14.17%	1.73%	321.1	17	93.40%	80.50%	8.60%	343.7
LE921	150 bp Paired	12.56%	1.61%	409	22	93.70%	80.50%	8.90%	436.3
LE922	150 bp Paired	13.18%	1.65%	435.6	23	93.40%	80.40%	8.80%	466.5
LEA01	150 bp Paired	13.13%	2.54%	459.5	24	93.70%	79.00%	9.30%	490.6
LEV01	150 bp Paired	13.66%	2.58%	502.7	26	93.90%	78.50%	9.60%	535.6

These statistics are based on the combined number of reads over all lanes of sequencing. * Note, this is assuming a genome size of 2.7Gbp^17^.

### Variant calling and filtering

Variant calling was performed using a parallel version of the freebayes software on the CSC Puhti server (Table 2) (CSC – IT Center for Science, Finland). Freebayes is a haplotype-aware variant caller and identified a total of 78,968,182 variants across the genome. After the application of various filters (Table 2) we recovered 11,363,883 high quality variants (Table 4) spread across the entire pseudoreference genome.

**Table 4:** Number of variants called and filtered.

Filter applied	Number of variants in	Number of variants out
Maximum missing genotype 50% -- Minor allele count 3 -- Minimum quality score 30.	78268182	19643329
Minor Allele Frequency 0.05 -- Minimum mean depth of 20	19643329	12813212
Filtering based on Allele Balance (AB): AB > 0.25 & AB < 0.75 | AB < 0.01	12813212	12260751
max mean depth of 30	12260751	11363883

### Mitochondrial genome assemblies

We assembled near complete mitochondrial genome sequences for each individual. The mitochondrial genome assemblies range from 16,648 bp to 17,612 bps in length, with four individuals producing circular contigs (LeA01, LE911, LE919 and LE921). All mitochondrial genes are present for each individual. The alignment of the mitochondrial genomes produced in this study are included as part of the Dryad dataset in the phylip format^23^.

## Technical Validation

**Fig. 2:**
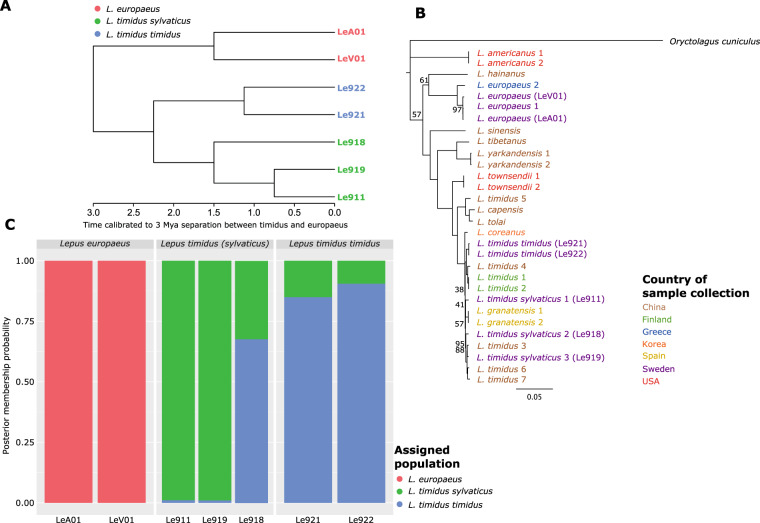
(**A**) Ultrametricised phylogenetic tree based on the genome wide single nucleotide variants of the samples. Node support <100 is reported. The phylogeny is calibrated to an estimated three million year separation between *L. europaeus* and *L. timidus*^41^. (**B**) Maximum likelihood tree of whole mitochondrial genomes of several *Lepus* species. The tree is rooted on *Oryctolagus cuniculus*. Tip labels are coloured based on the country of sample collection. Node support <100 is reported. (**C**) Complot analysis of the hare populations, showing the posterior membership probability of each hare sample. Note the differentiation between the two mountain hare subspecies.

The PCA analysis (Fig. 1B) and complot (Fig. 2C) of the filtered SNP dataset further confirms the different species groupings. A similar grouping pattern has been observed in *Lepus* species sampled throughout Finland^38^, where the genetic diversity of the *L. europaeus* is small, likely due to a founder effect resulting in tight clustering of these samples, with more broad clustering of the *L. timidus* samples (Fig. 1B). Interspecific introgression may also influence the observed patterns^38^. Of the 11,363,883 high quality variants, 1,230,035 were unique to the *L. europaeus* and 596,291 to *L. timidus* (Fig. 1C).

To confirm the relationship of the hare samples, we generated a maximum likelihood (ML) phylogenetic tree based on the whole genome SNPs (Fig. 2A). The phylogenetic clustering of these samples confirms the origin of the samples, as the first branch separates the *Lepus europaeus* and *Lepus timidus*, followed by the separation of the *L. timidus* and the subspecies *L. timidus sylvaticus*. The sub-species creates a further split between the sample from the mainland and the two collected on the islands in the southwest of Sweden. Similarly, the phylogeny based on the mitochondrial genomes (Fig. 2B), first shows a split between the *L. europaeus* and *L. timidus* samples. Then the *L. timidus timidus* and *L. timidus sylvaticus* samples were further split. Interestingly, with the inclusion of other *L. timidus* genomes from the far East, the *L. timidus sylvaticus* clustered with these samples. The observed differences between whole genome and mitochondrial genome data may be influenced by introgressive patterns as well as different means of inheritance.

## Usage Notes

The genomic data described could be used to assess adaptations of hares in, for example, pigmentation, cold durability and pathogen resistance in local, regional or a wide range of distributions. A particular value is to develop an integrated monitoring tool for assessing the conservation status of the heath hare in southern and central Sweden. This could be done by integrating genetic data, harvest data, pellet inventories and camera trapping. In addition to general conservation, the long-term preservation of the heath hare, as well as understanding of the nature of interactions between mountain hares and brown hares, is important from a resource perspective as hares are a game species in Sweden and elsewhere and, as such, a source of meat and recreation.

Despite the small sample set, the data indicates a notable number of private alleles in the two mountain hare subspecies (Fig. 1D). The resulting divergence between the two clades (Fig. 2A,B) is clearly visible in the phylogenies and in ancestry complot analysis (Fig. 2C), which support that the subspecies represent biological entities beyond a single gene winter pelage colour difference^39^. Furthermore, these private alleles could be used to examine the diversification of hares and their subspecies through the use of SNP panels. The subspecies split in Fig. 2A may have persisted in sympatry since the end of the last glacial maximum^40^, although the timing needs further validation^40^. The mechanisms maintaining the genetic division warrant further studies and raise an interesting question about the *bona fide* subspecies status of the heath hare.

**Fig. 3:**
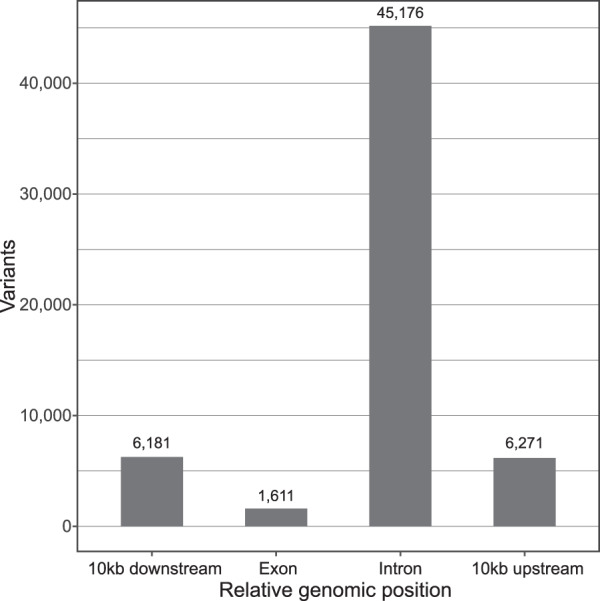
The number of Single Nucleotide Variants in and around known genes responsible for coat colour in mice.

To illustrate an example of how this genomic data could be integrated with future analyses we counted the number of variants found in known pigmentation genes. Although, the sample size for this analysis is too small in itself to provide any conclusions, we were able to identify a number of variants within and around genes (Fig. 3) with known effects on pigmentation^10,39^. If integrated with a larger dataset, we trust that the genomic data provided here will provide a valuable resource for population genetics, conservation biology and evolutionary studies on hares.

## Data Availability

All programs and commands used to present this data are contained in Table 2.
